# Selecting synaptic partners: GRASPing the role of UNC-6/netrin

**DOI:** 10.1186/1741-7007-9-43

**Published:** 2011-06-10

**Authors:** QueeLim Ch'ng

**Affiliations:** 1 MRC Centre for Developmental Neurobiology, New Hunt's House, Guy's Campus, King's College London, London SE1 1UL, UK

## Abstract

Forming synaptic connections of the appropriate strength between specific neurons is crucial for constructing neural circuits to control behavior. A recent paper in *Neural Development *describes the use of a synapse-specific label in *Caenorhabditis elegans *to implicate local UNC-6/netrin signaling in this developmental process. Thus, as well as their well known roles in cell migration and axon guidance, UNC-6/netrin signals distinguish an appropriate synaptic partner from other potential targets.

See Research article: http://www.neuraldevelopment.com/content/6/1/28

## 

Constructing neural circuits to perform biological functions is an intricate multi-step process that includes generating neurons, moving them to the right location, ensuring that they send out axons and dendrites to certain destinations, delivering pre- and postsynaptic components to the right parts of these neuronal outgrowths, and finally making the appropriate synaptic connections of the correct strength.

Specificity is crucial throughout this developmental process. In the final step of circuit formation, when axons or dendrites reach their target region, they are likely to be confronted with many potential synaptic partners within a relatively small space, from which they need choose the correct one. As circuit construction occurs in the context of the complex developing nervous system, multiple steps in the formation of multiple circuits might overlap in time and space, posing an additional requirement that distinct circuits do not cross-interfere. That neurons robustly form synapses with the correct partner, while avoiding unsuitable targets, is therefore especially important to ensure correct information transmission and eliminate inappropriate crosstalk between circuits.

What are the signals that govern this level of synapse specificity? A paper from Miri VanHoven and colleagues in *Neural Development *(Park *et al*. [1]) adroitly exploits the nematode *Caenorhabditis elegans *to identify UNC-6/netrin as a signal that controls the specific connectivity between two neurons. Notably, they applied a new labeling technique known as GFP reconstitution across synaptic partners (GRASP), which efficiently identifies specific synaptic contacts in living animals [2].

## Combining GRASP and behavioral analysis

**Figure 1:**
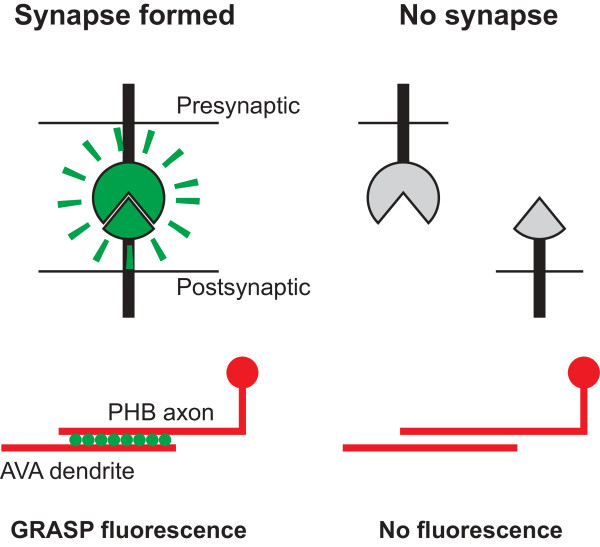
**The GRASP assay for detecting synapse formation between specific neurons**. Top: the two halves of split GFP are localized to pre- and postsynaptic specializations, respectively. Only when a synapse is formed will the complementary parts of GFP come close enough to reconstitute a fluorescent molecule (left). Bottom: GRASP used as a readout for synapse formation between the PHB and AVA neurons. Fluorescent puncta are only detected when synapses are formed between PHB and AVA (left).

Park *et al*. report the first application of the GRASP technique for identifying molecules that influence synaptic partner choice (Figure 1). In GRASP, green fluorescent protein (GFP) is split into two complementary parts, each of which is non-fluorescent on its own. One part is expressed in the presynaptic neuron and the other in the postsynaptic neuron; they are targeted to the extracellular pre- and postsynaptic specializations, respectively, by fusing them to NLG-1/neuroligin, a transmembrane synaptic protein. When the pre- and postsynaptic specializations come into close contact, the two complementary parts of GFP come together, like a handshake across the synapse, to reconstitute a fluorescent molecule (Figure 1, left). Thus, only the synapses between the selected pre- and postsynaptic neurons are visualized; other synapses on both partners remain unlabeled.

With thousands of synapses to choose from in *C. elegans*, Park *et al*. thoughtfully applied GRASP to the study of the extensive connections between a presynaptic sensory neuron (PHB) and its postsynaptic interneuron (AVA) (Figure 1, bottom). These connections are associated with a specific behavior: the inhibition of posterior movement in response to repellents sensed by PHB in the nematode tail [3].

Using GRASP-labeled PHB-AVA synapses to facilitate a screen of candidate mutants, the authors identified UNC-6/netrin and its receptor UNC-40/DCC as central players in the formation of this synaptic connection. They also find that a mutant in UNC-40/DCC shows the predicted increase in posterior movement consistent with reduced transmission between PHB and AVA, thus providing a behavioral correlate to structural synaptic changes in living animals to demonstrate biological relevance [1].

## Connecting with the right target

The number of PHB-AVA synaptic contacts is reduced in UNC-6/netrin or UNC-40/DCC mutants, whereas overexpression of UNC-6/netrin increases their number [1]. Together with the correlated behavioral analysis, these results of Park *et al*. indicate that the graded strength of synaptic connections can be controlled by UNC-6/netrin levels. Bidirectional control of graded transmission across two neurons also occurs during neuronal plasticity, raising the question as to whether proteins such as UNC-6/netrin are also involved in synaptic plasticity after the circuit is constructed.

Synaptic partner recognition utilizes short-range UNC-6/netrin signaling, which differs from the gradient models proposed for its roles in axon guidance. This short-range action is reminiscent of the role of netrin A/B in synaptic partner choice in the developing *Drosophila *neuromuscular junction [4], suggesting that the UNC-6/netrin system for synaptic partner recognition is likely to be conserved in other species as well. It will be interesting to determine whether other signals cooperate with or antagonize UNC-6/netrin in *C. elegans *(as those from semaphorin and immunoglobulin-like cell-adhesion molecules do in *Drosophila *[4]). In the absence of spatiotemporal mechanisms for separating potential targets, requirements for distinct molecules that signal unique target identity would be expected to increase as the density and number of potential targets increases. It will therefore be interesting to determine if combinatorial mechanisms that reduce the need for additional signals are used more frequently in more complex nervous systems or in more complex areas of a nervous system in a given species.

## Variations on UNC-6/netrin signaling and implications for neural development

UNC-6/netrin first gained prominence as an axon guidance molecule [5,6]; additional studies have revealed multifaceted roles for this guidance system, including in cell migration [5] and in glia-mediated coordination of synapse formation [7]. Park *et al*. [1] now show that UNC-6/netrin also acts as a direct signal for synaptic partner choice. The repeated use of the same signal in different processes raises interesting questions and implications for neuronal circuit development.

First, how does the same signal lead to different biological outcomes? The work of Park *et al*. reveals that several downstream effectors of UNC-40/DCC are not essential for synaptic partner recognition even though they play important roles in axon guidance [1]. Perhaps the UNC-6/netrin-UNC-40/DCC system has different modes of signaling that activate different effectors, leading to different outcomes. At the molecular level, do differences in ligand concentration, ligand presentation and the composition of receptor complexes have a role? Would these molecular differences separate short- and long-range modes of UNC-6/netrin signaling? At the cellular level, does specificity arise by signaling to only specific parts of the neuron, such as regions of axon competent for synapse formation (as suggested by the specific localization of UNC-40/DCC in the PHB axon [1]), and does the developmental state of the receiving neuron matter?

Second, how is neural development compartmentalized such that early signals do not influence late steps, and that the development of one circuit does not interfere with another nearby? Signaling specificity is likely to require a combination of several developmental and molecular mechanisms. UNC-6/netrin is deployed in different cells for guiding PHB axon outgrowth versus synapse formation [1,8]. Thus, spatial factors, despite the diffusible nature of UNC-6/netrin, are likely to play a role in minimizing cross-interference between different steps in neural development. Turnover of active signaling molecules would have to be fast relative to the time between different developmental steps, so that the old signal dies down before the neuron is ready to interpret a new signal for a different step. And perhaps different steps might be separated by different activation thresholds for distinct cell biological programs. Combinatorial regulation with additional signals, as seen in synapse formation in the *Drosophila *neuromuscular junction [4], might also provide an additional layer of specificity. Understanding the mechanisms that ensure robust, specific development in highly intricate nervous systems would be a major step in developmental neurobiology.

Finally, would the repeated use of the same molecule require developmental programs to minimize cross-interference and thus constrain the types of structures and anatomy that could develop? Or would it be an economical way of coordinating complex structures so that related structures are organized around a single cue? An integrated analysis of the multiple processes involved in neural circuit formation will be an important next step in addressing these big picture questions in development, and tools like GRASP [2] are very likely to play a prominent part in these future discoveries. The signaling molecules involved are highly conserved across the animal kingdom [4-6]; thus, the principles and mechanisms that emerge from studies in one organism can tell us much about the process in other species.
